# Modulating the phases of iron carbide nanoparticles: from a perspective of interfering with the carbon penetration of Fe@Fe_3_O_4_ by selectively adsorbed halide ions†
†Electronic supplementary information (ESI) available. See DOI: 10.1039/c6sc01819j



**DOI:** 10.1039/c6sc01819j

**Published:** 2016-08-18

**Authors:** Ziyu Yang, Tianshan Zhao, Xiaoxiao Huang, Xin Chu, Tianyu Tang, Yanmin Ju, Qian Wang, Yanglong Hou, Song Gao

**Affiliations:** a Department of Materials Science and Engineering , College of Engineering , Peking University , Beijing 100871 , China . Email: hou@pku.edu.cn; b Center for Applied Physics and Technology , College of Engineering , Peking University , Beijing 100871 , China; c College of Chemistry and Molecular Engineering , Peking University , Beijing 100871 , China

## Abstract

Iron carbide nanoparticles (ICNPs) are considered to have great potential in new energy conversion, nanomagnets and biomedical applications due to their intrinsically peculiar magnetic and catalytic properties. However, the synthetic routes were greatly limited in morphology and phase controlled synthesis. In this article, we present a versatile solution chemistry route towards colloidal ICNPs (Fe_2_C-hexagonal and monoclinic syngony, Fe_5_C_2_-monoclinic syngony and Fe_3_C-orthorhombic syngony) derived from body centered cubic Fe@Fe_3_O_4_ by introducing heteroatoms to restrain their phase transformation. We found that the phases of Fe_2_C NPs could be controlled by direct phase transformation in the drastic thermally driven procedure (defined as thermodynamical manner). Meanwhile, the selective adsorption of Cl ions weakened the bonding between Fe and C atoms, thus interfering with the penetration of C atoms to form lower carbon content Fe_5_C_2_ and Fe_3_C NPs. The kinetic mechanisms were evaluated using density functional theory (DFT) simulations focusing on the bonding energy between Fe–C and Fe–Cl atoms. All the obtained ICNPs exhibited typically soft ferromagnetic properties with the highest saturation magnetization value of 101.2 emu g^–1^ and the highest Curie temperature of 497.8 K.

## Abstract 2

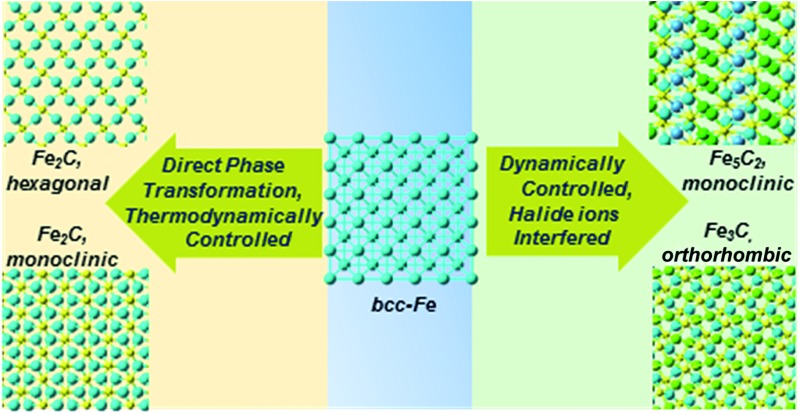
The phase controlled synthesis of iron carbide nanoparticles was proposed through a thermodynamical and dynamical manner by introducing hetero-halide ions.

## Introduction

Iron based magnetic nanomaterials have gained intense interest both theoretically and practically in the last two decades, and are perfect alternatives for various platforms from biomedical applications to environmental catalysis.^1,2^ Among which, most work has been carried out on oxides and metallic iron. Although iron oxides are both air/water stable, and nontoxic in organisms, the saturation magnetization is low, especially at the nanoscale. Moreover, metallic iron nanostructures possess poor stability when exposed to an oxygen/water-containing atmosphere, which precludes their wider applications.^3^ Iron carbides (ICs), primarily consisting of carbon atoms occupying interstitial sites of the iron lattice, constitute an attractive alternative with high saturation magnetization (*ca.* 140 emu g^–1^), high chemical stability, and favourable bio-compatibility compared to oxides and metallic iron.^4–6^ Thus, ICs are promising multifunctional platforms for biomedical applications, such as bio-imaging, drug delivery, photo-thermal therapy and hyperthermia.^7,8^ In addition, ICs were reported to be potential magnetic storage media, and presented excellent catalytic activities in resource and electrochemical energy conversion, such as Fischer-Tropsch synthesis (FTS) and the oxygen reduction reaction.^9–12^

Despite the intriguing advantages of IC nanostructures, the synthetic routes were greatly limited in the morphology and phase controlled synthesis, which was probably due to their intrinsically ‘harsh’ properties. Until now, few reports have concerned their synthesis in the form of nanostructures. The major preparation routes of ICs could be generalized to solid state reactions, laser ablation methods, sono-chemical and bio-templated based methods, yet aggregations and polydisperse particles were unavoidable in the final products.^13–18^ Solution chemistry routes, in which the generation and growth processes of particles are modulated by burst nucleation of the nucleus and precise supply of reactive entities, are important for the control of the size, shape, self-assembly and crystallinity of the nanostructures.^1^ Chaudret *et al.* reported the synthesis of near monodisperse Fe_2.2_C/Fe_5_C_2_ hybrid NPs utilizing seeded growth inspired by the FTS process.^19^ Besides, Fe_3_C NPs were also reported to be generated in ionic liquids [C_*n*_-MIM][BF_4_].^20^ Previously, our group reported the synthesis of single phase Fe_5_C_2_ NPs and evaluated their special catalytic activities in the FTS reaction.^10^ However, the phase and morphology control of ICNPs remains a great challenge due to the complicated phase diagram of Fe–C and uncontrolled growth.

Generally, crystalline ICs are formed through the ‘penetration’ process of carbon atoms. The formation of ICs with different stoichiometry of Fe–C could be explained by their relative thermodynamic stability; exploring the process of their phase transformations in a both thermodynamically and kinetically controlled manner is important.^11^ Halide ions were demonstrated to influence the shapes of noble metal nanocrystals due to their varied binding energy on different metal facets.^21^ For another example, halide ions were also used in thermal decomposition of Fe(CO)_5_ to generate crystalline Fe NPs with average size distributions and robust stability in air, in which the strong bonding of Cl^–^ and Fe induced the slow growth kinetics that was more thermodynamically stable.^22^ Despite all the distinct work reported, the function of halide ions was limited to the single-phase system. In fact, the peculiar selective abilities of halide ions to the metal surface make them a perfect platform for the modulation of ‘conversion chemistry’ from a special metal. How to introduce halide ions in the phase transformation system, and to explore their operational mechanism is interesting and significant.

Herein, we report a facile and versatile solution chemistry synthesis of colloidal ICNPs of tuned phases and controlled morphologies by introducing hetero-halide ions. The synthetic procedures were conducted in a ‘seed-conversion’ manner; Fe_2_C (hexagonal, monoclinic syngony), Fe_5_C_2_ (monoclinic syngony) and Fe_3_C (orthorhombic syngony) colloidal ICNPs could be obtained. Besides, the effects of halide ions on the formation process were simulated based on density functional theory (DFT). To our knowledge, it is the first time that ICNPs have been obtained in one system with tuned phases and growth kinetics.

## Results and discussion

**Fig. 1:**
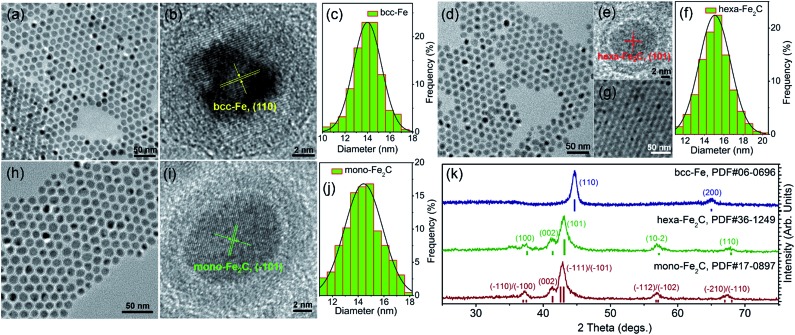
Morphological and structural analysis of bcc-Fe@Fe_3_O_4_ and Fe_2_C NPs. (a–c) TEM, HRTEM images and the statistical histogram of the 14.0 ± 0.8 nm bcc-Fe@Fe_3_O_4_ NPs. (d–f) TEM, HRTEM images and the statistical histogram of the 14.9 ± 0.8 nm hexa-Fe_2_C NPs. Fig. 1g shows the multilayer hexa-Fe_2_C NPs assemblies acquired *via* solvent evaporation. (h–j) TEM, HRTEM images and the statistical histogram of the 15.0 ± 0.8 nm mono-Fe_2_C NPs. (k) PXRD patterns of the bcc-Fe@Fe_3_O_4_ and Fe_2_C NPs.

The transmission electron microscopy (TEM) image showed that the decomposition of Fe(CO)_5_ in the presence of NH_4_Br produced spherical body centered cubic Fe (bcc-Fe) NPs with a homogeneous Fe_3_O_4_ shell, exhibiting a mean diameter of 14.0 ± 0.8 nm, as shown in Fig. 1a. The crystal structure of the as-synthesized Fe@Fe_3_O_4_ was confirmed by powder X-ray diffraction (PXRD) studies (see Fig. 1k). No peaks of Fe_3_O_4_ were observed except for two strong peaks corresponding to the (110) and (200) facets of bcc-Fe, which was due to the peak broadening of their small and weak crystal domains of the Fe_3_O_4_ shell. The contrast in the high resolution transmission electron microscopy (HRTEM) image also revealed the core–shell structure (see Fig. 1b). In the typical ‘conversion’ step, the bcc-Fe NPs were transformed to a four-neck bottle containing octadecanamine (ODA), and then the mixture was programmed to elevated temperatures under a N_2_ blanket. It is notable that after the carburization procedure, the statistical diameter of the final as-synthesized NPs was expanded due to the lattice distortion caused by carbon atom penetration, with an average diameter of 14.9 ± 0.8 nm, as shown in Fig. 1d–g. The HRTEM image of a single as-synthesized nanoparticle is shown in Fig. 1e, indicating a lattice fringe of 0.21 (3) nm that was characteristic of the (101) planes of hexagonal Fe_2_C (hexa-Fe_2_C) NPs. The shell was composed of amorphous entities with several tiny crystalline domains that represent an inverse spinel structured iron oxide Fe_3_O_4_. PXRD results carried out on powder samples showed the appearance of five peaks that confirm the carbon-rich IC phase hexa-Fe_2_C with the space group, *P*6_3_/*mmc* (194). Interestingly, a void space clearly existed between the Fe_2_C core and Fe_3_O_4_ shell, which acted as the possible reactive area in the carbon penetration process, indicating the Kirkendall effect through the lattice transition.^23^ Besides, when the programmed temperature was higher than 290 °C, monoclinic carbon-rich carbide mono-Fe_2_C was obtained, with a diameter distribution of 15.0 ± 0.8 (Fig. 1h–j) and exposing a diffraction fringe of 0.21 (3) nm indicating the (–101) facets (Fig. 1i). The PXRD result revealed that the main peaks of the mono-Fe_2_C (–101) and (–111) facets (PDF#17-0897, space group void) shifted to small angles compared to hexa-Fe_2_C NPs (PDF#36-1249), indicating heavier lattice distortions compared to the initial bcc-Fe (space group *Im*3*m*, 229) symmetry, as shown in Fig. 1k. Interestingly, bcc-Fe and mono-Fe_2_C could also be acquired when using trimethylamine-*N*-oxide (TMAO) as the oxidative agent to form Fe_3_O_4_ shells (see Fig. S1, ESI†).

**Fig. 2:**
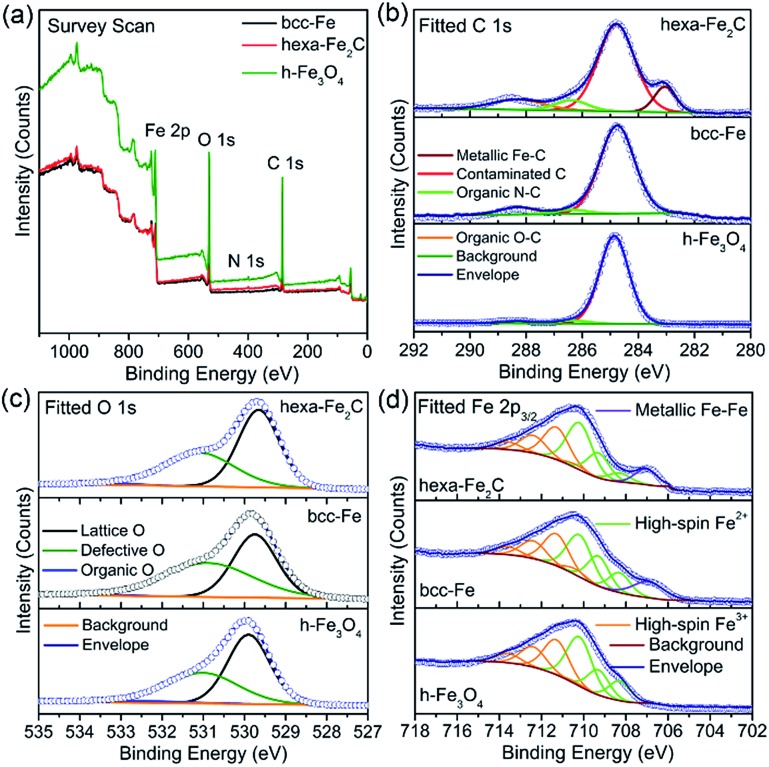
(a–d) Survey and fitted X-ray photoelectron spectrums (XPS) of hexa-Fe_2_C, bcc-Fe and Fe_3_O_4_ NPs. (a) Survey scan. (b) C 1s region. (c) O 1s region and (d) Fe 2p_3/2_ region fitted using Gupta–Sen multiplets.

Benefiting from the highly surface-sensitive properties of XPS, the changes in the photoelectron peak shape indicating special chemical states could be acquired. In our research, samples of bcc-Fe, hexa-Fe_2_C and inverse spinel structured hollow Fe_3_O_4_ (h-Fe_3_O_4_, see Fig. S2, ESI†) for parallel samples were characterized with XPS analysis. All the charging of the samples was controlled by using a charge neutralizer filament, and calibrated using the adventitious C 1s peak with a fixed value of 284.8 eV. Fig. 2a shows the characteristic photoelectron peaks of the as-synthesized NPs with scan binding energy (BE) from 0 to 1100 eV, no additional elements were detected except for the core or outer orbital level photo-electrons and Auger electrons of the Fe, C, N and O elements. The C 1s core level spectrums are shown in Fig. 2b, with the primary peak at 284.8 eV attributable to the surface contaminated carbon and peaks at 286.4 eV and 288.3 eV defined as the organic N–C and O–C species. Notably, a strong low-BE peak signal at 283.1 eV was detected that represented the typical metallic Fe–C bonding, which was similar to the previously reported Ni_3_C compounds.^24^ However, peaks around 283.1 eV were not detected in the spectrums of metallic bcc-Fe and h-Fe_3_O_4_ NPs, confirming the strong Fe–C interactions in the ICs. Besides, for the O 1s region (Fig. 2c), peaks at 529.7 eV, 531.1 eV and 533.3 eV were typically near to the lattice O and defective O sites due to the high ratio of surfaces defects, and organic O species, respectively.^25–27^

A Shirley background-subtracted Fe 2p_3/2_ spectrum is characterized in Fig. 2d, indicating the existence of multivalent iron oxide Fe_3_O_4_ and metallic Fe bonding. It is notable that a thick Fe 2p_3/2_ envelope with a shoulder on the low-BE side was specific to hexa-Fe_2_C (707.1 eV) and bcc-Fe (707.0 eV), compared to the h-Fe_3_O_4_ NPs, which was ascribed to the statistical Fe–C and metallic Fe bonding. The high-BE shift of metallic Fe to Fe–C bonding spectrum was probably due to the electrostatic interactions, spin–orbit coupling between the 2p core hole and unpaired 3d electrons of the photo-ionized Fe cations and Fe–C crystal field interactions. Besides, the Fe 2p_3/2_ spectrum could be well fitted using the Gupta–Sen multiplets for both the Fe^2+^ and Fe^3+^ components with a low-BE ‘pre-peak’ introduced for high-spin Fe^2+^ portions.^28,29^

**Fig. 3:**
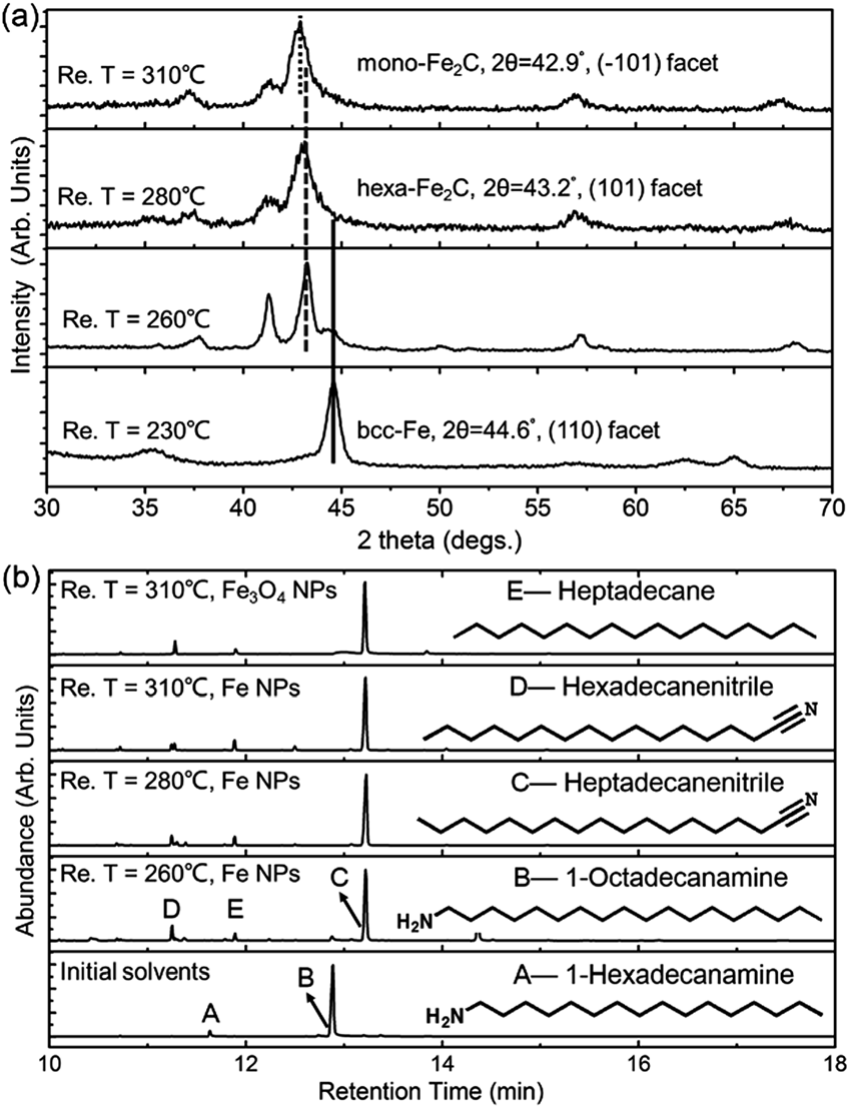
PXRD and GC-MS data for the isolated samples and solvents. (a) PXRD data for the isolated samples extracted at 230 °C, 260 °C, 280 °C, and 310 °C. (b) GC-MS for the isolated solvents extracted with bcc-Fe and Fe_3_O_4_ NPs as catalysts at different temperatures.

To determine the formation process of the FeC_2_ NPs, the near *in situ* products were examined using PXRD and GC-MS. PXRD patterns of the intermediate samples extracted at reaction temperature (Re. T) of 230 °C, 260 °C, 280 °C, and 310 °C are shown in Fig. 3a. The diffraction profiles were indexed as bcc-Fe even at a Re. T of 230 °C, but with the appearance of hexa-Fe_2_C with (101) facets when the Re. T reached 260 °C (the compound was of 15% wt Fe and 85% wt Fe_2_C, calculated from PXRD profiles). Then the main peak positions showed progressive shift to lower angles as the Re. T increased to higher than 260 °C, which was consistent with the expansion of the unit cell as the interstitial carbon atoms were incorporated (bcc-Fe, 2*θ* = 44.6°, (110) facet, to hexa-Fe_2_C, 2*θ* = 43.2°, (101) facet). The hexa-Fe_2_C NPs were fully formed at the Re. T of 280 °C. Interestingly, with the Re. T increased to 310 °C, mono-Fe_2_C NPs were obtained. The transformation was ascribed to a thermodynamic change with heavy lattice distortions due to a drastic thermally driven shift (from cubic symmetry to monoclinic symmetry, with the main peak positions shifted to 2*θ* = 42.9°, 42.5°, (–101) and (–111) facets). To explore the transformation from hexa-Fe_2_C to mono-Fe_2_C NPs, we introduced PXRD characterizations of samples extracted at an average temperature interval of 15 °C, and further verified the gradual shifting process. Interestingly, no distinct crystal formation was observed upon increasing the Re. T to higher temperatures even at 345 °C, indicating that Fe_2_C phases were thermally favourable during the lattice transitions (see Fig. S3a, ESI†).

In modern organic transformations catalyzed by transition metals, iron and iron salts have been found to have high activities towards carbon–carbon and carbon–heteroatom bond-forming reactions.^30–32^ Jana *et al.* reported an efficient iron-catalyzed C–C single-bond cleavage *via* retro-Claisen condensation, which was a mild and convenient approach to synthesize various esters and ketones.^33^ To fully understand the catalytic mechanism in the carbonization process, we introduced GC-MS and FT-IR studies to identify the intermediate reactions. As shown in Fig. 3b, the initial solvents were mainly composed of 1-octadecanamine with 1-hexadecanamine impurities. When the Re. T increased to 260 °C and higher temperatures, peaks of 1-octadecanamine and 1-hexadecanamine disappeared with the appearance of peaks of 1-heptadecanenitrile and 1-hexadecanenitrile, indicating the transformation of –NH_2_ to –C≡N. Moreover, in addition to 1-heptadecanenitrile and 1-hexadecanenitrile, plenty of saturated hydrocarbons such as heptadecane were detected which could be ascribed to the C–C cleavage of the nitriles. Meanwhile, the formed free C species acted as the carbon source. To verify the catalytic sites in the decomposition process, we synthesized h-Fe_3_O_4_ in the approximate size distribution of the Fe@Fe_3_O_4_ seeds, and conducted the synthetic process in the same procedure (see Fig. S2, ESI†). In the GC-MS results extracted at the Re. T of 310 °C in the contrast experiment, 1-heptadecanenitrile, 1-hexadecanenitrile and heptadecane were detected, indicating that Fe_3_O_4_ was an efficient catalyst for the C–C cleavage. The FT-IR characterizations further confirmed the proposed hypothesis above (see Fig. S3b, ESI†).

**Fig. 4:**
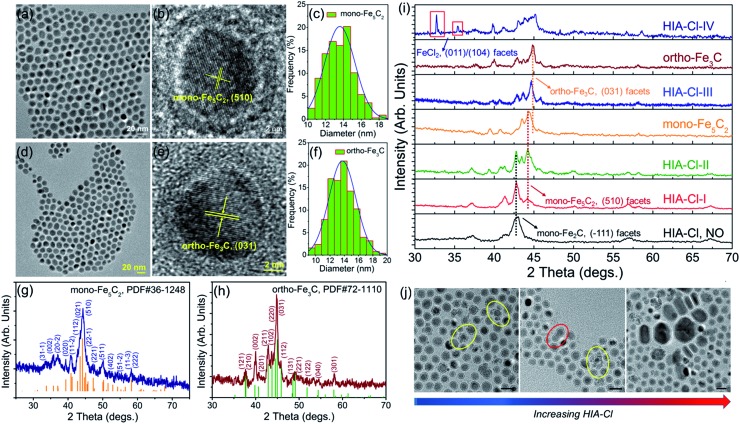
Morphological and structural analysis of mono-Fe_5_C_2_ and ortho-Fe_3_C NPs. (a) and (b) TEM and HRTEM images of the 14.3 ± 0.8 nm mono-Fe_5_C_2_ NPs. (c) Size distribution histogram of mono-Fe_5_C_2_ NPs. (d–f) TEM, HRTEM images and the size distribution histogram of the 14.1 ± 0.8 nm ortho-Fe_3_C NPs. (g) and (h) PXRD patterns of mono-Fe_5_C_2_ and ortho-Fe_3_C NPs, with shifted diffraction angles of 44.2° (mono-Fe_5_C_2_) and 44.9° (ortho-Fe_3_C) compared to bcc-Fe. (i and j) Artificial monitoring of phase and morphological evolution of ICNPs: (i) PXRD patterns indicating the phase transformation process, with shifted diffraction angles of the main peaks. (j) TEM images showing the morphological evolution with increasing HIA-Cl, particles in circles showing the two kinds of morphological evolution process, collapse of the shell (yellow) and fusion of the two isolated NPs (red). The scale bars are 20 nm.

Different from the synthesis of hexa-Fe_2_C and mono-Fe_2_C NPs, monoclinic Fe_5_C_2_ (mono-Fe_5_C_2_) and orthorhombic Fe_3_C (ortho-Fe_3_C) NPs were acquired through a ‘dynamically controlled’ manner. In the typical synthesis utilizing cetyltrimethyl ammonium chloride (CTAC) as the halide ion inducer, similar spherical ICNPs were obtained. TEM and HRTEM images of mono-Fe_5_C_2_ are shown in Fig. 4a and b. The NPs were in an average diameter distribution of 14.3 ± 0.8 nm, with an evident void between the core carbide phase and the coating Fe_3_O_4_ shell. Interestingly, the ortho-Fe_3_C NPs (average diameter distribution of 14.1 ± 0.8 nm) could be obtained by increasing the additional halide ions systematically (see Fig. 4d–f). PXRD profiles were in accordance with mono-Fe_5_C_2_ (PDF#36-1248, with an impurity of 3% wt Fe_2_C, calculated by PXRD profiles) and ortho-Fe_3_C (PDF#72-1110, with an impurity of 2% wt Fe_5_C_2_), respectively, as shown in Fig. 4g and h. To further explore their surface properties, we introduced XPS analysis to reveal the Fe–C and Fe–O bonding, as shown in Fig. S4, ESI.† The Brunauer–Emmett–Teller (BET) analysis utilizing nitrogen sorption isotherms is shown in Fig. S5, ESI.†

Previously, Pd nanocubes with slight truncation at the corners and edges and Pd–Pt bimetallic nanocrystals were prepared using chemisorbed Br ions; the halide ions were introduced to ensure selective deposition of atoms onto the corner sites during seed-mediated growth.^34^ Besides, in a study by Sun *et al.*, Cl ions were proposed as an agent that efficiently inhibited the Fe growth kinetics.^35^ To determine the roles that halide ions acted in the ICNPs formation process, we monitored the phase and morphology evolutions by tuning the reaction parameters systematically. By varying the additional amount of halide ions (taking CTAC as a typical example, denoted as HIA-Cl-I to HIA-Cl-IV, with mole ratios of CTAC : Fe of 0.003 : 1, 0.01 : 1, 0.03 : 1, and 0.05 : 1, respectively), the structures went through heavy lattice distortions from cubic to monoclinic and orthorhombic syngony. PXRD profiles of the performed particles showed that the crystalline phase was mono-Fe_2_C if without HIA-Cl. However, when the HIA-Cl was increased, the mono-Fe_2_C acted as the primary phase and gradually vanished with the appearance of the mono-Fe_5_C_2_ phase. The phase ratio of HIA-Cl-I to HIA-Cl-III was of 47% wt Fe_2_C : 53% wt Fe_5_C_2_, 17% wt Fe_2_C : 83% wt Fe_5_C_2_, and 57% wt Fe_5_C_2_ : 43% wt Fe_3_C, respectively. The complete disappearance of the mono-Fe_2_C phase initiated when the HIA-Cl was around 0.02 : 1, and finally transformed to the ortho-Fe_3_C phase when the HIA-Cl was around 0.04 : 1 (see Fig. 4i). If the HIA-Cl was introduced higher than 0.05 : 1 (denoted as HIA-Cl-IV), a slight amount of FeCl_2_ phase was detected, indicating the strong bonding ability of Fe–Cl. Different from the transformation of hexa-Fe_2_C to mono-Fe_2_C phases, we hypothesize that the chemical adsorption of hetero-Cl ions to the Fe_3_O_4_ shell interferes with the carbon penetration paths, thus making the carbon atoms’ diffusion difficult, *i.e.* the halide ions make the phase transformation occur in a kinetically controlled manner. From a perspective of carbon penetration, the ‘amount’ of carbon atoms was restrained by halide ions during the seed-mediated growth, so that a lesser ratio of carbon atoms in the ortho-Fe_3_C phase was obtained. TEM images of the ICNPs synthesized with increasing HIA-Cl are shown in Fig. 4j. It was observed that the protective Fe_3_O_4_ shell was easily collapsed and crushed during the heating process. The destruction of the protective Fe_3_O_4_ shell went through two processes: collapse and erosion, and finally resulted in uncontrolled or second growth of large particles. Interestingly, we found that the strong affinity surfactant trioctylphosphine oxide (TOPO) or oleyl alcohol as co-surfactants were sufficient to avoid the collapse and crushing of the final products. This may arise from the robust chemical adsorption due to the strong affinity abilities of –P=O and –OH to the Fe_3_O_4_ shell.^36,37^

**Fig. 5:**
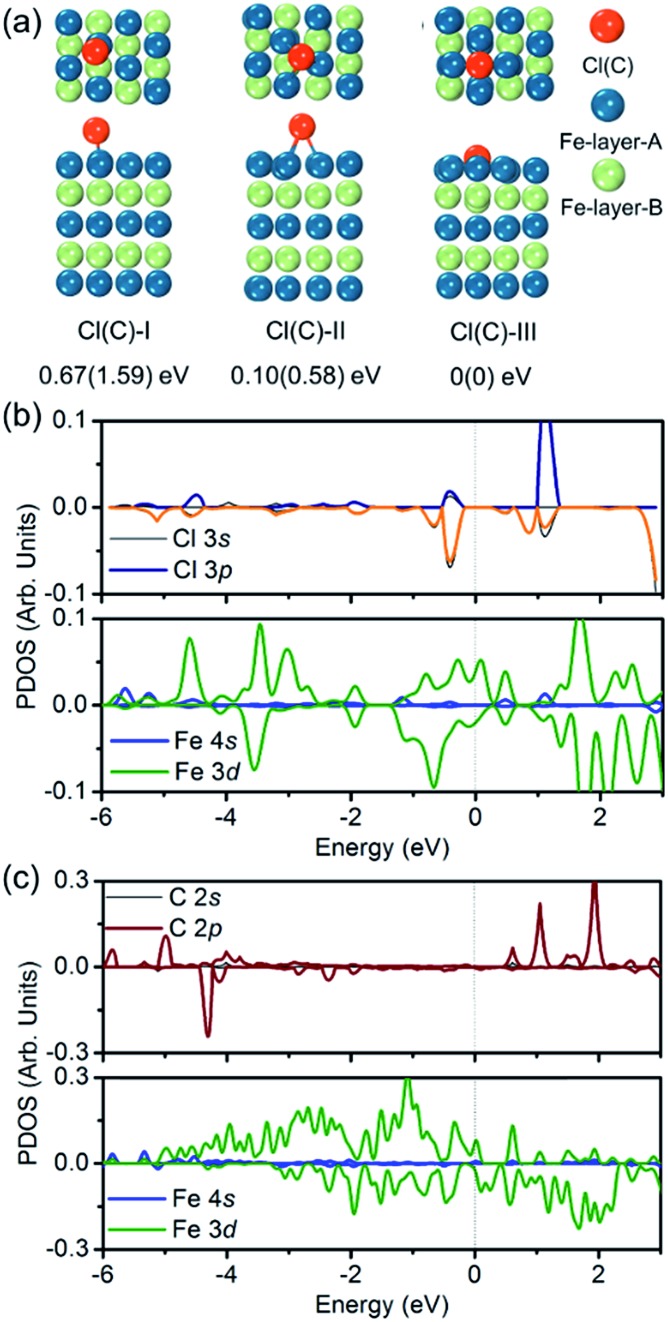
(a) Top and side views of three adsorption configurations of a Cl(C) atom on the Fe (101) surface, and the relative energies with respect to the most stable configuration III – (I) top-site, (II) bridge-site, and (III) hollow-site. (b and c) Partial DOS of the most stable configuration of the Cl(C) adsorbed Fe (101) surface.

To verify the hypothesis above, we constructed a simulation model to evaluate the role Cl ions acted, focused on the affinity abilities. Based on the space symmetry of the Fe (101) surface, three highly possible adsorption configurations were considered for a single Cl(C) atom: configuration Cl(C)-I, where the Cl(C) atom was on the top-site of the Fe atom, configuration Cl(C)-II, where the Cl(C) atom was on the bridge-site of the Fe atoms, and configuration Cl(C)-III, where Cl(C) was on the hollow site of the Fe atoms (see Fig. 5a). Geometry optimization and total energy calculations revealed that the hollow site was energetically most favorable. Configuration Cl(C)-III was lower in energy by 0.67(1.59) eV and 0.10(0.58) eV than configuration Cl(C)-I and Cl(C)-II for Cl(C) adsorption, respectively, indicating that the adsorption of Cl(C) on the Fe surface was site-dependent. In the lowest energy configuration, the average distance of Cl(C) to the four neighboring Fe atoms was 2.41(1.87) Å, and the adsorption energy of C was 0.36 eV lower than that of Cl, indicating that the adsorption of C was energetically more favorable than that of Cl on the Fe surface. Meanwhile, for both configurations Cl(C)-I and Cl(C)-II, the adsorption of the Cl atom was more energetically favorable than that of C. Based on the Bader charge analysis, we found that the average C atom received 1.33 electrons and the Cl atom received 1.05 electrons from the four neighboring Fe atoms in configuration Cl(C)-III. Due to the fact that the atomic size of Cl is larger than that of C, which gives rise to a longer Fe–Cl bond length compared to that of Fe–C, the bonding was weaker and the charge transfer was less in Fe–Cl, although the electron affinity of Cl was larger than that of C.

We then calculated the partial density of states (PDOS) of Fe atoms that bonded with the Cl(C) atom for the lowest energy configuration, as presented in Fig. 5b and c. It was indicated that the magnetic moment was mainly contributed by the Fe 3d orbitals in both the Fe–Cl and Fe–C systems. The Cl 3p orbitals in the spin up and spin down channels located around –0.5 eV were the bonding states with the Fe 3d orbitals. Meanwhile, the C 2p orbitals in the spin up channel located around –6 eV and –5 eV were the bonding states with the Fe 3d orbitals. The C 2p orbitals in the spin down channel located around 4.30 eV were the anti-bonding state with the Fe 3d orbitals. Comparing the PDOS of Cl with C, it was noted that the C 2p states were deeper than the Cl 3p states in both the spin up and spin down channels.

**Fig. 6:**
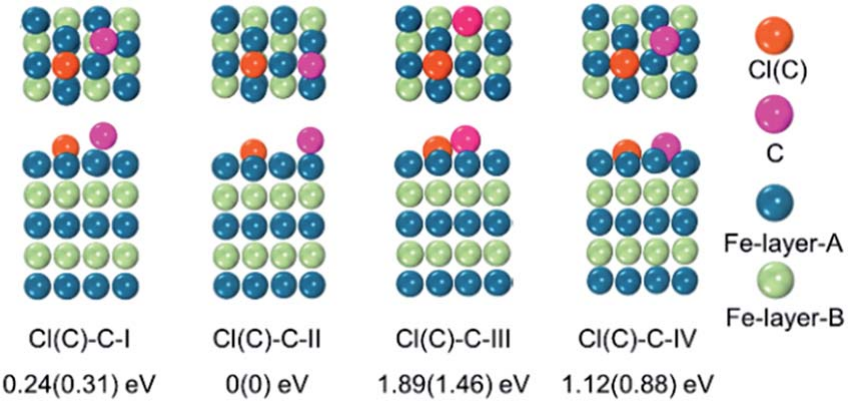
Top and side views of the adsorption configurations of the second C on the Cl(C) adsorbed Fe (101) surface, and their relative energies with respect to the most stable configuration II – (I) second C on Fe surface hollow-site-A, in which C–Cl(C) distance is 3.07(2.87) Å, (II) second C on Fe surface hollow-site-B, in which C–Cl(C) distance is 3.95(3.94) Å, (III) top-site, and (IV) bridge-site.

To examine whether the adsorption of Cl inhibited the adsorption of the C atom on the Fe surface, we introduced an additional C atom into the Cl(C) adsorbed Fe surfaces to explore the most preferable site of the second C atom. Starting with the most preferable configuration of the C(Cl) adsorbed Fe (101) surface, as shown in Cl(C)-III (see Fig. 5a), the second C atom was at the four nonequivalent sites, as shown in Fig. 6. The most energetically preferable configuration was found to be Cl(C)–C-II, where the second C atom was on the hollow site of the Fe atoms in both the Cl and C adsorbed Fe surfaces. This configuration was 0.24(0.31) eV, 1.89(1.46) eV, and 1.12(0.88) eV lower in energy than configuration I, III, and IV, respectively. More importantly, we found that in this lowest energy configuration, the adsorption energy of the second C on the Cl adsorbed Fe surface was 0.20 eV less than that on the C adsorbed Fe surface. It was suggested that the existence of Cl on the Fe surface weakened the bonding between the C and Fe atoms, thus restraining the adsorption of C atoms, qualitatively in good agreement with our experimental findings. Hence, the modulation of ICNPs is defined as two different pathways: (1) a direct phase transformation in a thermodynamically controlled manner, in which C atoms penetrate due to the drastic thermally driven process to form Fe_2_C NPs; (2) a dynamically controlled manner, where the selectively adsorbed Cl ions weaken the bonding between Fe and C atoms, thus interfering with the absorption of C atoms, and forming lower carbon content Fe_5_C_2_ and Fe_3_C NPs.

**Fig. 7:**
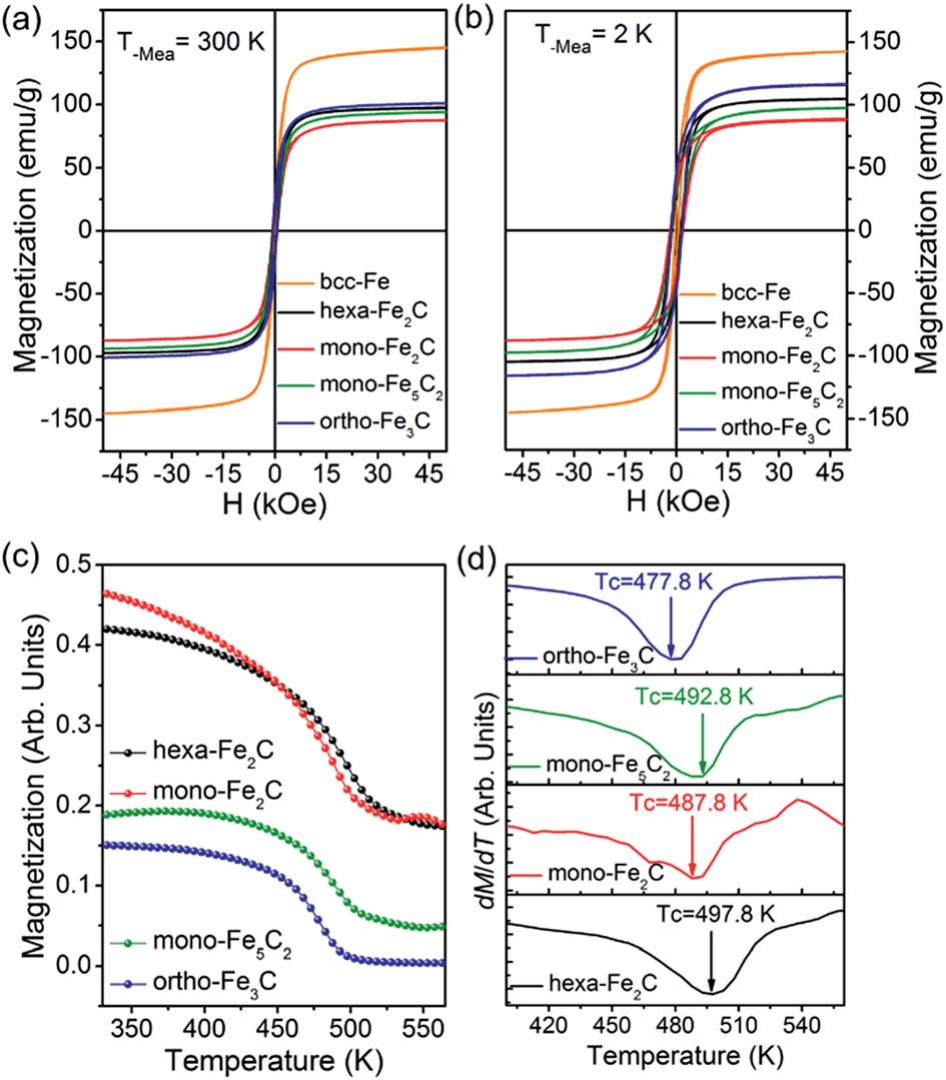
Magnetic properties of the initial bcc-Fe and the preformed ICNPs. (a and b) *M vs. H* curves at 300 K and 2 K, respectively. (c and d) *M vs. T* and the d*M*/d*T* curves indicating the Curie temperatures.

The magnetization measurements were investigated *via M*–*H* loops with an applied magnetic field of 5 T and *M*–*T* curves in the temperature range 300–560 K. All the samples were purified and sealed in plastic capsules before characterization. The absolute magnetization value was deduced from Fe^(0)^ elemental content determined using an inductively coupled plasma atomic emission spectrometer (ICP-AES). As was expected, all the initial bcc-Fe and performed ICNPs exhibited soft ferromagnetic behaviour at room temperature and 2 K, as shown in Fig. 7a and b. It was noticed that the *M*_s_ and *H*_c_ values were slightly higher at 2 K than at 300 K, which could be interpreted by considering the thermal activation of the nano-structured materials. The measured saturation magnetization (*M*_s_) of the initial bcc-Fe was 144.5 emu g^–1^, with a coercivity of 189.2 Oe, lower than the bulk entities (220 emu g^–1^), which was due to the size effect and the ferrimagnetic Fe_3_O_4_ shell. All the ICNPs exhibited lower *M*_s_ than that of the bcc-Fe, which was in accordance with the theoretical simulations that strong Fe–C bonding reduced the local magnetic moment of the Fe atoms.^38^

The ortho-Fe_3_C NPs possessed the highest *M*_s_ values of the ICNPs, around 101.2 emu g^–1^, with a coercivity of 543.9 Oe, still lower than the bulk entities due to the size effects in the nanoscale.^6^ Interestingly, the hexa-Fe_2_C exhibited higher *M*_s_ values (97.9 emu g^–1^, 495.4 Oe) than mono-Fe_2_C NPs (88.2 emu g^–1^, 653.7 Oe). The mono-Fe_5_C_2_ possessed an *M*_s_ value of 95.0 emu g^–1^, and a coercivity of 636.5 Oe, which was lower than our previous report due to the smaller size.^8^ The *M*–*T* curves were measured using a vibrating sample magnetometer (VSM) with an applied probe field of 100 mT (see Fig. 7c). The Curie temperature (*T*_c_) could be defined as the maximum slope in d*M*/d*T* deduced from a special field, as shown in Fig. 7d, all the ICNPs exhibited a *T*_c_ around 490 K, with the highest *T*_c_ of hexa-Fe_2_C (497.8 K), and the lowest *T*_c_ of ortho-Fe_3_C (477.8 K). The detailed magnetic measurement results are shown in Table 1.

**Table 1:** Detailed magnetic measurement results

Sample	*M*_s_/emu g^–1^ (300 K/2 K)	*H*_c_/Oe (300 K/2 K)	*T*_c_/K	Size/nm
bcc-Fe	144.5/144.2	189.2/537.9	—	14.0 ± 0.8
hexa-Fe_2_C	97.9/105.2	495.4/1305.5	497.8	14.9 ± 0.8
mono-Fe_2_C	88.2/88.5	653.7/1617.5	487.8	15.0 ± 0.8
mono-Fe_5_C_2_	95.0/98.0	636.5/1520.5	492.8	14.3 ± 0.8
ortho-Fe_3_C	101.2/116.7	543.9/1574.5	477.8	14.1 ± 0.8

## Conclusions

In summary, we proposed a versatile solution method for the phase controlled synthesis of various colloidal ICNPs by introducing hetero-halide ions, with crystalline geometry from hexagonal (Fe_2_C) to monoclinic (Fe_2_C, Fe_5_C_2_) and orthorhombic (Fe_3_C). It was the first time that ICNPs were obtained in one system with tuned phases and growth kinetics. The phase transformation mechanisms could be defined in a thermodynamically controlled and kinetically controlled manner. The selective adsorption of hetero-halide ions was essential in the synthesis of mono-Fe_5_C_2_ and ortho-Fe_3_C. Based on DFT simulations, we found that by selectively adsorbing Cl ions on the Fe surface, the C penetrating process was interfered with, thus restraining the additional C atoms into the Fe lattice to form lower carbon content ICNPs. All the preformed ICNPs expressed a typical soft ferromagnetic characterization with the highest *M*_s_ value of 101.2 emu g^–1^, and the highest *T*_c_ of 497.8 K. The proposed synthetic route based on the thermodynamic and kinetic control of the ‘seed-conversion’ method can be extended to other synthetic categories, especially in the synthesis of transition metal carbide nanostructures. Moreover, we present a generally new strategy to modulate the crystalline phase by introducing heteroatoms to restrain or accelerate the conversion process.

## Experimental

### Synthesis of bcc-Fe NPs as seeds

In the typical synthesis, 62.5 mmol ODE, 0.1 mmol NH_4_Br and 1 mmol OAm were mixed magnetically and degassed under a gentle N_2_ flow for 1 h in a four-neck flask. The solution was then heated to 100 °C and kept at this temperature for 2 h before it was heated further to 180 °C to fully remove the organic impurities. After that, 5 mmol Fe(CO)_5_ was injected to the reaction mixture and kept there for 30 min. A color change from salmon to brown then to black of the solvents were observed after *ca.* 1 min, indicating the decomposition of Fe(CO)_5_ and the formation of bcc-Fe NPs.

To control the oxidation of the as-prepared bcc-Fe NPs, 1 mmol OA and hexane (0.2 ml) were added *via* a syringe and the resultant solution was aged at 140 °C for another 30 min before it was cooled down to room temperature. Acetone (27 ml) was added to precipitate the product. After centrifugation (11 min, 11 500 g), the product was collected and re-dispersed in hexane and precipitated using ethanol. The washing procedure was repeated three times and the final product, 14.0 ± 0.8 nm bcc-Fe NPs, was dispersed in methylbenzene with several droplets of OAm for further carbonization.

### Synthesis of ICNPs using bcc-Fe NPs as seeds

Typically, Fe_2_C NPs were synthesized as follows: ODA (37.5 mmol) or a mixture of ODA (11.5 mmol) and OAm (15.5 mmol) was magnetically blended in a four-neck flask and degassed under a gentle N_2_ flow for 1 h at 120 °C. Then, the resulted bcc-Fe NPs (5 mmol, in 10 ml hexane) were added *via* a syringe and the reaction solution was heated at 130 °C for another 30 min to remove hexane thoroughly before it was heated to the target temperature (260–345 °C) for 15–45 min. The black-brown colored solution was cooled down to room temperature by removing the heating source. Acetone (27 ml) was added and the mixture was centrifuged (11 min, 11 500 g). The precipitates were purified with hexane (15 ml) and ethanol (20 ml) three times before being dispersed in methylbenzene (10 ml) with several droplets of OAm. Fe_5_C_2_ and Fe_3_C NPs were obtained in the same procedure except for the addition of halide entities (CTAC or NH_4_Br) and TOPO (0.05–0.07 mmol), or oleyl alcohol (8–12 mmol): Fe_5_C_2_ NPs–NH_4_Br (0.03–0.05 mmol), or CTAC (0.11–0.13 mmol); Fe_3_C NPs–NH_4_Cl (0.11–0.13 mmol), CTAC (0.17–0.21 mmol).

### Simulation methods and models

Our first principles calculations within the framework of density functional theory (DFT) were carried out using Vienna *Ab initio* Simulation Package (VASP).^39^ The exchange-correction potential was treated by using the generalized gradient approximation (GGA) in the form proposed by Perdew, Burke, and Ernzerhof (PBE).^40^ A plane-wave basis set with the projector augmented plane-wave (PAW) method was used to calculate the total energies and forces, and to optimize the geometries.^41^ We have modeled the surface of bcc-Fe having the (101) orientation by a slab consisting of five atomic layers with a (2 × 2) supercell containing 40 Fe atoms in the A-B-A-B-A stacking pattern. Each slab was separated from the other by a vacuum region of 15 Å, and the iron atoms on the bottom three layers were held at their bulk configuration while those on the upper two layers were allowed to relax without any symmetry constraint. The reciprocal spaces were represented by Monkhorst–Pack special *k*-points of 7 × 7 × 1 meshes. The 2s^2^2p^2^, 3s^2^3p^5^, and 3d^6^4s^2^ atomic orbitals were treated as the valence states for C, Cl, and Fe, respectively.^42^ Because the GGA could not properly describe the strongly correlated system containing partially filled d atoms, we used the GGA + U method by dividing the electrons into two classes: delocalized s and p electrons, which could be well described by the GGA, while the localized d electrons were described by the Coulomb and exchange corrections. We used a correlation energy (*U*) of 4.00 eV and an exchange energy (*J*) of 1.00 eV for Fe 3d orbitals.^43^ These values have been tested and widely used in previous studies.^44–46^ The energy cut-off was set to 500 eV, and the convergence in energy and force was 10^–4^ eV and 10^–2^ eV Å^–1^, respectively. Bader charge analysis was carried out to study the charge distribution and transfer quantitatively.^47^

## References

[cit1] Hyeon T. (2003). Chem. Commun..

[cit2] Yang W., Rehman S., Chu X., Hou Y., Gao S. (2015). ChemNanoMat.

[cit3] Huber D. L. (2005). Small.

[cit4] Giordano C., Kraupner A., Wimbush S. C., Antonietti M. (2010). Small.

[cit5] Giordano C., Antonietti M. (2011). Nano Today.

[cit6] Hofer L., Cohn E. M. (1959). J. Am. Chem. Soc..

[cit7] Cowger T. A., Tang W., Zhen Z., Hu K., Rink D. E., Todd T. J., Wang G. D., Zhang W., Chen H., Xie J. (2015). Theranostics.

[cit8] Yu J., Yang C., Li J., Ding Y., Zhang L., Yousaf M. Z., Lin J., Pang R., Wei L., Xu L. (2014). Adv. Mater..

[cit9] Zhao D., Shui J.-L., Chen C., Chen X., Reprogle B. M., Wang D., Liu D.-J. (2012). Chem. Sci..

[cit10] Yang C., Zhao H., Hou Y., Ma D. (2012). J. Am. Chem. Soc..

[cit11] de Smit E., Cinquini F., Beale A. M., Safonova O. V., van Beek W., Sautet P., Weckhuysen B. M. (2010). J. Am. Chem. Soc..

[cit12] Pumera M., Ambrosi A., Chng E. L. K. (2012). Chem. Sci..

[cit13] Hirano S.-I., Tajima S. (1990). J. Mater. Sci..

[cit14] Song H., Chen X. (2003). Chem. Phys. Lett..

[cit15] Schnepp Z., Wimbush S. C., Antonietti M., Giordano C. (2010). Chem. Mater..

[cit16] Schnepp Z., Yang W., Antonietti M., Giordano C. (2010). Angew. Chem., Int. Ed..

[cit17] Nikitenko S. I., Koltypin Y., Palchik O., Felner I., Xu X. N., Gedanken A. (2001). Angew. Chem., Int. Ed..

[cit18] Amendola V., Riello P., Meneghetti M. (2010). J. Phys. Chem. C.

[cit19] Meffre A., Mehdaoui B., Kelsen V., Fazzini P. F., Carrey J., Lachaize S., Respaud M., Chaudret B. (2012). Nano Lett..

[cit20] Lartigue L., Long J., Dumail X., Nikitenko S. I., Cau C., Guari Y., Stievano L., Sougrati M. T., Guérin C., Sangregorio C. (2013). J. Nanopart. Res..

[cit21] Xiong Y., Cai H., Wiley B. J., Wang J., Kim M. J., Xia Y. (2007). J. Am. Chem. Soc..

[cit22] Lacroix L.-M., Frey Huls N., Ho D., Sun X., Cheng K., Sun S. (2011). Nano Lett..

[cit23] Yin Y., Rioux R. M., Erdonmez C. K., Hughes S., Somorjai G. A., Alivisatos A. P. (2004). Science.

[cit24] Goto Y., Taniguchi K., Omata T., Otsuka-Yao-Matsuo S., Ohashi N., Ueda S., Yoshikawa H., Yamashita Y., Oohashi H., Kobayashi K. (2008). Chem. Mater..

[cit25] Biesinger M. C., Payne B. P., Grosvenor A. P., Lau L. W., Gerson A. R., Smart R. S. C. (2011). Appl. Surf. Sci..

[cit26] Grosvenor A., Kobe B., Biesinger M., McIntyre N. (2004). Surf. Interface Anal..

[cit27] Yamashita T., Hayes P. (2008). Appl. Surf. Sci..

[cit28] Gupta R., Sen S. (1975). Phys. Rev. B: Solid State.

[cit29] Gupta R., Sen S. (1974). Phys. Rev. B: Solid State.

[cit30] Zhou S., Junge K., Addis D., Das S., Beller M. (2009). Angew. Chem..

[cit31] Hatakeyama T., Nakamura M. (2007). J. Am. Chem. Soc..

[cit32] Enthaler S., Junge K., Beller M. (2008). Angew. Chem., Int. Ed..

[cit33] Biswas S., Maiti S., Jana U. (2010). Eur. J. Org. Chem..

[cit34] Xia X., Xie S., Liu M., Peng H.-C., Lu N., Wang J., Kim M. J., Xia Y. (2013). Proc. Natl. Acad. Sci..

[cit35] Zhang S., Jiang G., Filsinger G. T., Wu L., Zhu H., Lee J., Wu Z., Sun S. (2014). Nanoscale.

[cit36] Gao G., Liu X., Shi R., Zhou K., Shi Y., Ma R., Takayama-Muromachi E., Qiu G. (2010). Cryst. Growth Des..

[cit37] Caruntu D., Yao K., Zhang Z., Austin T., Zhou W., O'Connor C. J. (2010). J. Phys. Chem. C.

[cit38] Fang C. M., van Huis M. A., Zandbergen H. W. (2009). Phys. Rev. B: Condens. Matter Mater. Phys..

[cit39] Kresse G., Furthmüller J. (1996). Phys. Rev. B: Condens. Matter Mater. Phys..

[cit40] Perdew J. P., Burke K., Ernzerhof M. (1996). Phys. Rev. Lett..

[cit41] Blöchl P. E. (1994). Phys. Rev. B: Condens. Matter Mater. Phys..

[cit42] Monkhorst H. J., Pack J. D. (1976). Phys. Rev. B: Solid State.

[cit43] Anisimov V. I., Aryasetiawan F., Lichtenstein A. (1997). J. Phys.: Condens. Matter.

[cit44] Sato K., Bergqvist L., Kudrnovský J., Dederichs P. H., Eriksson O., Turek I., Sanyal B., Bouzerar G., Katayama-Yoshida H., Dinh V. (2010). Rev. Mod. Phys..

[cit45] Bernien M., Miguel J., Weis C., Ali M. E., Kurde J., Krumme B., Panchmatia P. M., Sanyal B., Piantek M., Srivastava P. (2009). Phys. Rev. Lett..

[cit46] Zhou J., Sun Q. (2011). J. Am. Chem. Soc..

[cit47] Tang W., Sanville E., Henkelman G. (2009). J. Phys.: Condens. Matter.

