# Supplementary material for: Modulating the phases of iron carbide nanoparticles: from a perspective of interfering with the carbon penetration of Fe@Fe3O4 by selectively adsorbed halide ions
Source: Chem Sci. 2016 Aug 18;8(1):473–81. doi: 10.1039/c6sc01819j (PMC5298209; doi:10.1039/c6sc01819j)
Supplement: Supplementary file 1 [file SC-008-C6SC01819J-s001.pdf]

Electronic Supplementary Material (ESI) for Chemical Science.
This journal is © The Royal Society of Chemistry 2016

Electronic Supplementary Information (ESI)

Modulating the Phases of Iron Carbide Nanoparticles: From a Perspective of Interfering the Carbon Penetration of Fe@Fe_3O_4 by Selectively Absorbed Halide Ions

Ziyu Yang,[a] Tianshan Zhao,[b] Xiaoxiao Huang,[a] Xin Chu,[a] Tianyu Tang,[a] Yanmin Ju,[a] Qian Wang,[b] Yanglong Hou[a] and Song Gao[c]*

[a] Department of Materials Science and Engineering, College of Engineering, Peking University, Beijing 100871, P. R. China. Email: hou@pku.edu.cn

[b] Center for Applied Physics and Technology, College of Engineering, Peking University, Beijing 100871, P. R. China.

[c] College of Chemistry and Molecular Engineering, Peking University, Beijing 100871, China.

The electronic supplementary information contains the following sections:

Experimental Section

1. *Chemicals.*
2. *Characterizations.*

Supplementary Figures

Fig. S1-S5

References

Experimental Section

1. Chemicals

All the chemicals were used without additional purification, and were performed under argon utilizing a homemade heating apparatus, four-neck bottles (Synthware), a glove box (MIKROUNA) and Ar/N_2/vacuum lines.

Octadecene (ODE, tech. 90%), Trioctyl-phosphine Oxide (TOPO, 98%), NH_4Cl (99.5%), Cetanecyl Trimethyl Ammonium Chloride (CTAC, 96%) were purchased from Alfa Asear. Octadecanamine (ODA, 90%), NH_4Br (99%) were purchased from J&K Chemicals. Oleylamine (OAm, tech. 70 %), Oleic Acid (OA, 99%), Trimethylamine N-Oxide (TMAO, 98%) were purchased from Sigma Aldrich. Iron Pentacarbonyl were from Tianyi Co. Ltd, Jiangsu, China.

2. Characterization

The conventional bright-field images were performed utilizing FEI Tecnai T20 microscope (200 kV), and high-resolution TEM (HRTEM) was carried out on an FEI Tecnai F30 microscope (300 kV). Powder X-ray diffraction (PXRD) patterns were obtained using a PANalytical X'Pert^3 Powder X-ray diffractometer equipped with Cu-Kα radiation at 40 kV and 40 mA, respectively. X-ray photoelectron spectroscopy (XPS) measurements were performed on Imaging Photoelectron Spectrometer (Axis Ultra DLD, Kratos Analytical Ltd.) using monochromatized Al Kα anode (Al Ka, $h\nu$=1486.7 eV), along with the pressures in the analysis chamber of 10^{-8}-10^{-9} torr. All the collected spectra were calibrated with contaminated C 1s peak at 284.8 eV, and were analyzed using CasaXPS software (2.3.12 Dev7). Fourier Transform Infrared Spectrometer (FTIR) measurements were carried out on a Nicolet FTIR spectrometer (Magna-IR 750). The solvent extractions were studied by the Gas Chromatography−Mass Spectrometry (GC−MS) apparatus (Agilent 7890A GC with a 5975C mass-selective detector). Magnetic susceptibility data were collected with Physical Property Measurement System (PPMS-9, Quantum Design) working in the temperature range of 2–390 K and with magnetic field up to 5 T. M-T curves in the range of 300–550 K were measured with a VSM magnetometer (Model 4 HF VSM, ADE USA) with applied probe field of 100 mT. The absolute magnetization was calibrated from $Fe^{(0)}$ content determined by Inductively Coupled Plasma-Atomic Emission Spectrometer (PROFILE SPEC, Leeman). Nitrogen sorption isotherms were measured utilizing the Quantachrome Autosorb-IQ instrument at 77 K.

Supplementary Figures

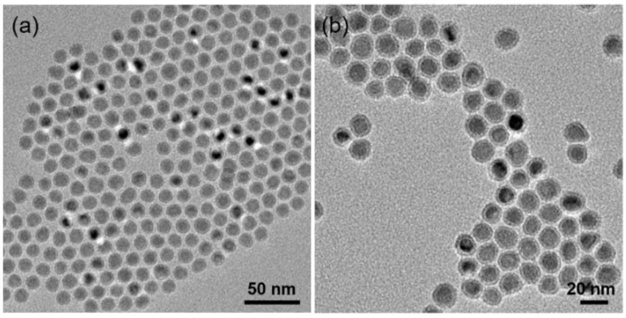

Fig. S1 (a) TEM images of the bcc-Fe oxidized with TMAO as the oxidative agent. (b) mono-Fe_2C.

In the typical synthesis of bcc-Fe@Fe_3O_4 using Trimethylamine N-Oxide (TMAO) as oxidative agent, 62.5 mmol ODE, NH_4Br (0.1 mmol) and 1 mmol OAm were mixed magnetically and degassed under a gentle N_2 flow for 1 h in a four-neck flask. The solution was then heated to 100 °C and kept at this temperature for 2 h before it was heated further to 180 °C to fully remove the organic impurities. After that, 5 mmol $Fe(CO)_5$ was injected to the reaction mixture and kept there for 30 min. When the temperature was cooled down to 140 ^{o}C, 0.1 mmol TMAO and etahnol (0.2 ml) were added via a syringe and the resultant solution was aged at 140 °C for another 30 min before it was cooled down to room temperature. It was clear that the obtained bcc-Fe@Fe_3O_4 was similar to the NPs that obtained using OA as the oxidative agent. The carbonization process was also similar to the procedures mentioned in the article.

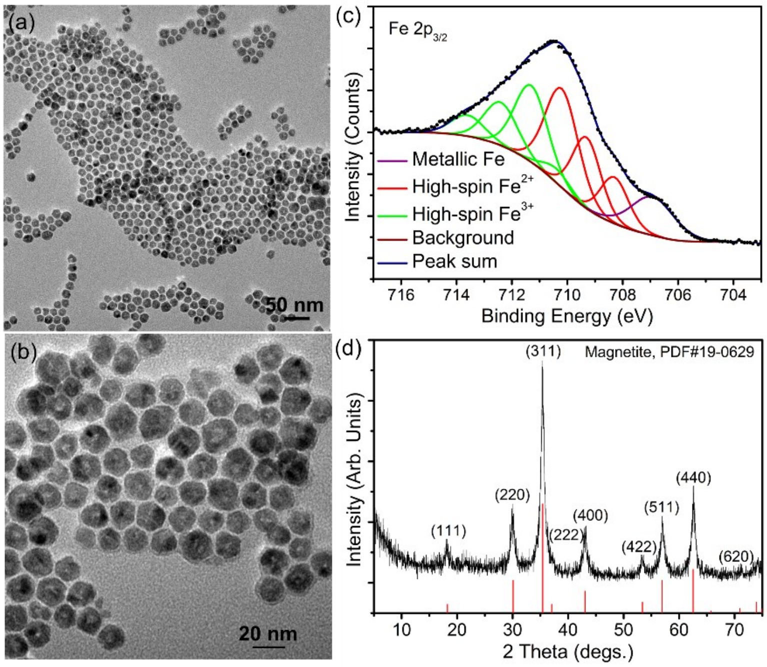

Fig. S2 Morphological and structural analysis of the hollow Fe_3O_4 (h-Fe_3O_4) NPs. (a-b) TEM images of the h-Fe_3O_4 NPs. (c) Fitted X-ray photoelectron spectra of the Fe $2p_{3/2}$ region using Gupta-Sen multiplets. (d) PXRD characterization of the h-Fe_3O_4 NPs.

The parallel samples h-Fe_3O_4 were characterized with PXRD and XPS (See Fig. S2). All the charging of the samples was controlled by using a charge neutralizer filament, and calibrated using the adventitious C 1s peak with a fixed value of 284.8 eV. The Shirley background-subtracted Fe $2p_{3/2}$ spectrum indicated that the parallel samples was of inverse spinel structured Fe_3O_4, and could be typically fitted using the Gupata-Sen multiplets for both the high-spin Fe^{2+} and Fe^{3+} components.[1, 2]

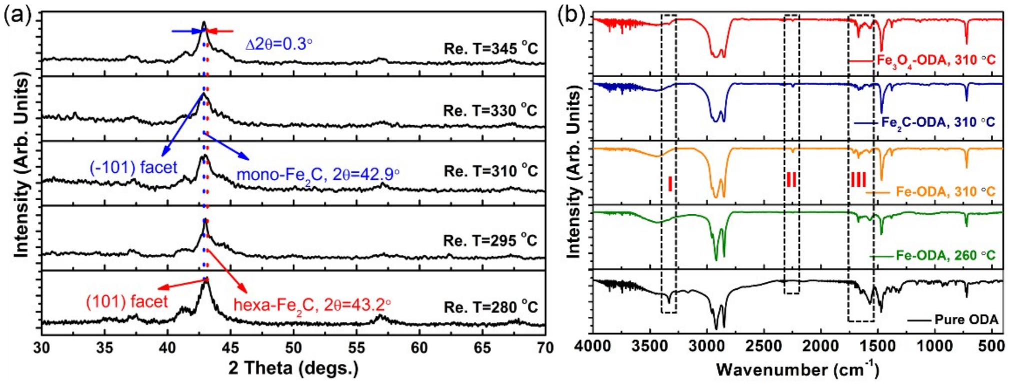

Fig. S3 (a) PXRD characterizations of samples extracted at an average temperature interval of 15 °C from 280 to 310 °C, indicating the gradual transformation of hexa-Fe_2C to mono-Fe_2C. When the temperature Re. T was higher than 310 °C and increased to 345 °C, the phases were kept mono-Fe_2C, showing that the Fe_2C phases were thermally favorable during the lattice transitions. (b) FTIR spectra of the reacting solvents extracted at different temperatures with bcc-Fe, mono-Fe_2C and h-Fe_3O_4 NPs of 260 °C, 310 °C and the original solvent. The frames indicated for the peaks were corresponding to the chemical bonds of $-NH_2$ (I), C≡N (II) and C=C (III).

To determine the transformation from hexa-Fe_2C to mono-Fe_2C NPs, we introduced PXRD characterizations of samples extracted at an average temperature interval of 15 °C, and further verified the gradual profiles shifting process. Interestingly, no distinct crystal formation was observed upon increasing the Re. T to higher temperatures, indicating that Fe_2C phases were thermally favorable during the lattice transitions (see Fig. S3a). The FTIR results further confirmed the GC-MS results mentioned above, as shown in Fig. S3b. With the increased Re. T, peaks of $-NH_2$ at around 3332 cm^{-1} vanished gradually and the peaks of C≡N around 2245 cm^{-1} appeared simultaneously.[3] Moreover, when using mono-Fe_2C and h-Fe_3O_4 as the catalytic agent, the similar peaks were observed, indicating that the surface Fe_3O_4 were the specific catalytic agent in the long-chain amines decomposing process.

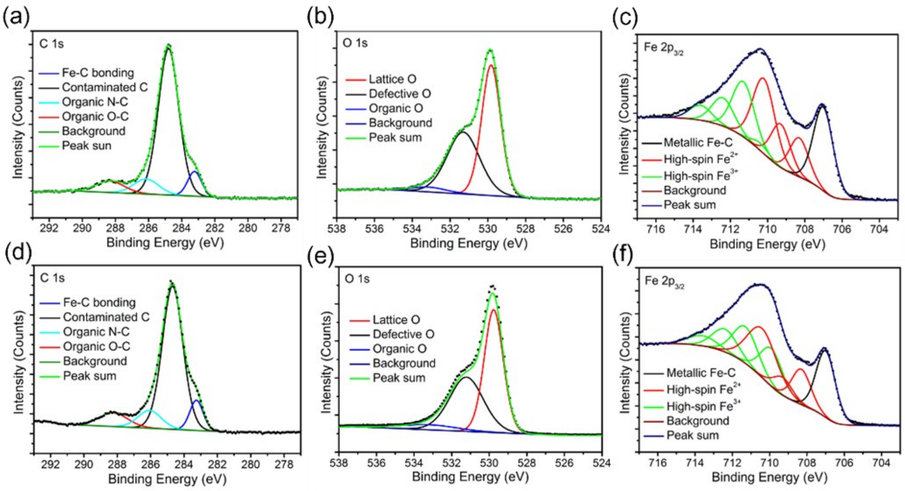

Fig. S4 The typical fitted C 1s, O1s and Fe $2p_{3/2}$ X-ray photoelectron spectrums of the mono-Fe_5C_2 and ortho-Fe_3C NPs. (a-c) mono-Fe_5C_2 NPs. (d-f) ortho-Fe_3C NPs.

In our research, all the charging of the samples was controlled by using a charge neutralizer filament, and calibrated using the adventitious C 1s peak with a fixed value of 284.8 eV. Fig. S4a-c showed the characteristic photoelectron peaks of the as-synthesized mono-Fe_5C_2 NPs. The C 1s core level spectrums were shown in Fig. S4a, with the primary peak at 284.8 eV attributable to the surface contaminated carbon and peaks at 286.2 eV and 288.4 eV defined as the organic N-C and O-C species. The strong low-BE peak signal at 283.4 eV was detected that represented the typical metallic Fe-C bonding, which was similarly to the previously reported Ni_3C compounds.[4] Besides, for the O 1s region (Fig. S4b), peaks at 529.7 eV, 531.1 eV and 533.3 eV were typically near to the lattice O, defective O sites due to the high ration of surfaces defects, and organic O species, respectively.[5-7] The characteristic peaks of ortho-Fe_3C was almost the same with mono-Fe_5C_2 (see Fig. S4d-e), except that the corresponding Fe-C peaks were at 283.3 eV.

A Shirley background-subtracted Fe $2p_{3/2}$ spectrum was characterized in Fig. S4c and 4f, indicating the existence of multivalent iron oxide Fe_3O_4 in both the mono-Fe_5C_2 and ortho-Fe_3C surface. All the Fe $2p_{3/2}$ spectrums could be well fitted using the Gupta-Sen (GS) multiplets for both the Fe^{2+} and Fe^{3+} components with a low-BE 'pre-peak' introduced for high-spin Fe^{2+} portions.[1, 2]

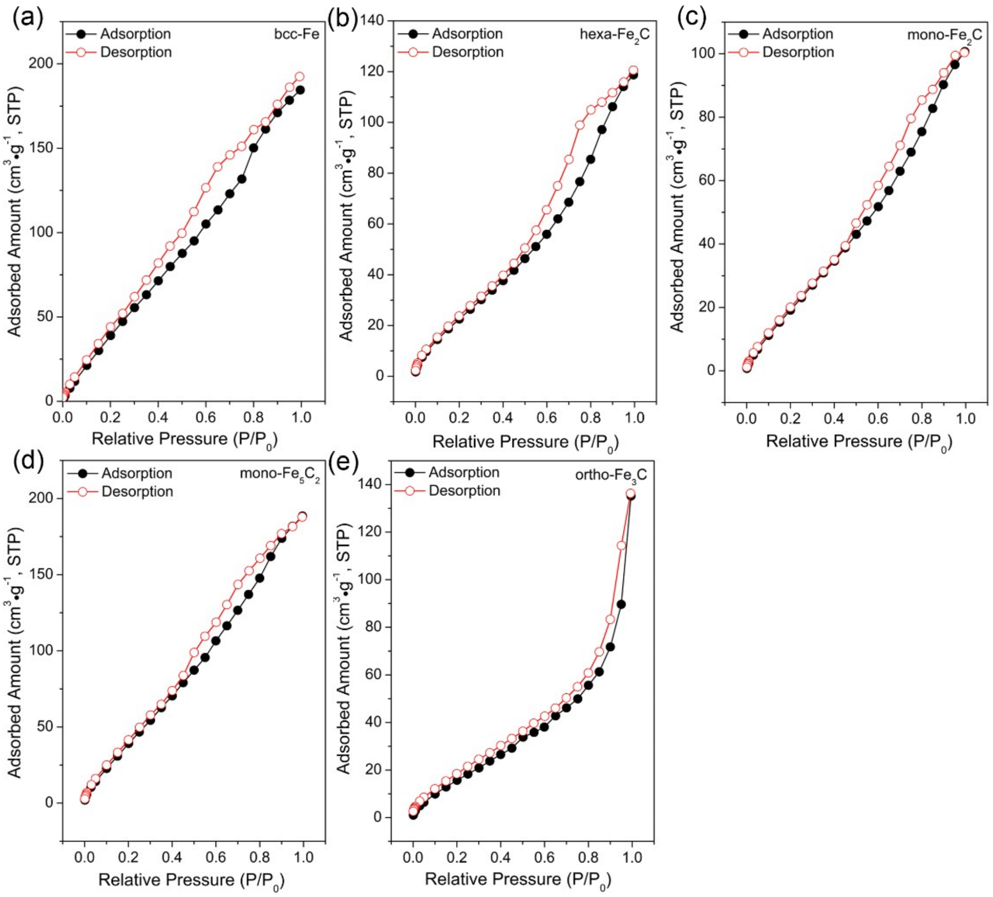

Fig. S5 Nitrogen sorption isotherms of the as-obtained nanoparticles. (a) bcc-Fe NPs. (b) hexa-Fe_2C NPs. (c) mono-Fe_2C NPs. (d) mono-Fe_5C_2 NPs. (e) ortho-Fe_3C NPs.

The BET analysis utilizing nitrogen sorption isotherms showed that all the nanoparticles were with high surface area of 243 $m^2 \cdot g^{-1}$ (bcc-Fe), 99 $m^2 \cdot g^{-1}$ (hexa-Fe_2C), 99 $m^2 \cdot g^{-1}$ (mono-Fe_2C), 191 $m^2 \cdot g^{-1}$ (mono-Fe_5C_2), and 71 $m^2 \cdot g^{-1}$ (ortho-Fe_3C), respectively, as shown in Fig. S5a-e.

References

1. R. Gupta and S. Sen, *Phys. Rev. B*, 1974, **10**, 71.
2. R. Gupta and S. Sen, *Phys. Rev. B*, 1975, **12**, 15.
3. C. Yang, H. Zhao, Y. Hou and D. Ma, *J. Am. Chem. Soc.*, 2012, **134**, 15814-15821.
4. Y. Goto, K. Taniguchi, T. Omata, S. Otsuka-Yao-Matsuo, N. Ohashi, S. Ueda, H. Yoshikawa, Y. Yamashita, H. Oohashi and K. Kobayashi, *Chem. Mater.*, 2008, **20**, 4156-4160.
5. M. C. Biesinger, B. P. Payne, A. P. Grosvenor, L. W. Lau, A. R. Gerson and R. S. C. Smart, *Appl. Surf.*
Sci., 2011, **257**, 2717-2730.
6. T. Yamashita and P. Hayes, *Appl. Surf. Sci.*, 2008, **254**, 2441-2449.
7. A. Grosvenor, B. Kobe, M. Biesinger and N. McIntyre, *Surf. Interface Anal.*, 2004, **36**, 1564-1574.
